# Lingering Pain and Discomfort Despite Splinting of an Ankle Fracture-Dislocation May Imply a Misdiagnosed Bosworth Injury: A Case Report

**DOI:** 10.7759/cureus.79358

**Published:** 2025-02-20

**Authors:** Efstratios D Athanaselis, Alexandros Saridis, Ioannis Nastas, Theofilos Karachalios, Sokratis Varitimidis

**Affiliations:** 1 Department of Orthopaedic Surgery and Musculoskeletal Trauma, University Hospital of Larissa, Larissa, GRC

**Keywords:** ankle fracture-dislocation, axilla sign, bosworth injury, distal tibiofibular syndesmosis, fibula dislocation, malleolar fracture

## Abstract

Bosworth fracture-dislocation is a challenging injury of the ankle highlighted by the entrapment of the proximal fibula segment behind the distal tibia. The rarity of these fractures frequently leads to unsuccessful closed reduction attempts, due to soft tissue interposition and locked dislocation of the fibula. We present a Bosworth injury initially misdiagnosed as a common Weber B lateral malleolus fracture. Increasing patient discomfort warrants a thorough re-evaluation of X-rays to identify the complexity of a Bosworth injury and ensure effective surgical treatment.

## Introduction

Bosworth fractures constitute a rare subtype, estimated at 1.6% of ankle injuries, first thoroughly stated by Bosworth in 1947 [1,2]. A distinct characteristic of these injuries is that the proximal fibula segment is irreducibly locked posteriorly, against the tubercle of the distal tibia as a result of supination and external rotation of the fibula (or internal rotation of the tibia, according to some authors) [3,4]. Due to their rarity among ankle fracture-dislocations, Bosworth injuries remain a diagnostic challenge, and the rate of misdiagnosis is high resulting in repetitive unsuccessful attempts of closed reduction, and intraoperative difficulties during open reduction, increasing tissue and cartilage damage [5,6]. A thorough examination of proper X-rays can establish Bosworth's injury diagnosis. Computed tomography (CT-scan) and three-dimensional reconstructions are valuable in understanding its complexity and successfully planning of surgical interventions [7].

This case report reviews recent presentations of Bosworth fracture-dislocations and states the highlights of diagnostic and therapeutic complexities along with the associated complications.

## Case presentation

**Figure 1:**
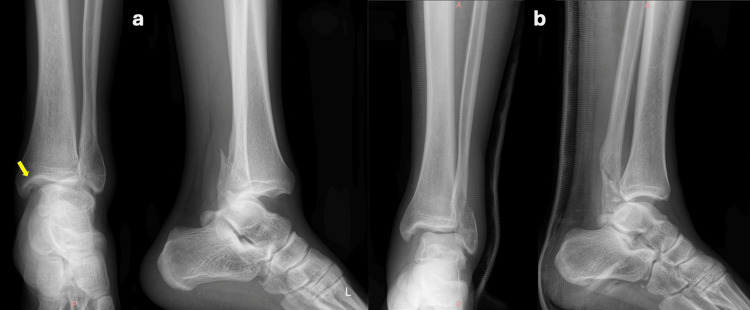
Pre-operative X-rays. (a) Initial anteroposterior and lateral x-rays revealing a left ankle joint dislocation with lateral malleolus fracture, unfortunately without forming a suspicion of a Bosworth injury (b) Closed reduction manipulations forced dislocated fibula to posterior angulation and failed to restore the anatomy causing increasing pain and discomfort to the patient in the next hours. The presence and the notability of the “axilla sign” (arrow) are controversial.

A 28-year-old male presented in our department with a left ankle injury during soccer. Oedema and deformity without neurovascular disorder were marked and the patient was unable to move his foot, holding it in an externally rotated position. After the radiological examination including anteroposterior and lateral views, a lateral malleolar ankle fracture-dislocation of type B according to the Danis-Weber classification, was revealed (Figure 1a). After multiple strenuous attempts at ankle reduction, it was immobilized in a splint, and new X-rays were taken; however, the ankle subluxation was underestimated (Figure 1b). The patient was admitted to the orthopaedic department for surgical treatment which was programmed for the next day.

Despite keeping the injured lower limb in an elevated, resting position, and medication including analgesics, NSAID, and corticosteroid injections for preventing blisters, the patient was persistently complaining about worsening pain and discomfort. Meticulous examination of the X-rays revealed the persistent dislocation of the fibula that was significantly posteriorly than its normal position relative to the tibia. The deformity could be clearly observed on the lateral X-ray while on anteroposterior view a Weber B lateral malleolar fracture overshadowed the controversial “axilla sign”. A Bosworth ankle fracture-dislocation was diagnosed (Figure 1b).

The patient was operated on under epidural anaesthesia and the Bosworth injury was confirmed. The fibula’s proximal segment was in an ectopic position deep in the posterior compartment of the leg making surgical exposure difficult. Moreover, disengaging the displaced fibula was exceptionally difficult, clearly demonstrating the "locked fibula" as described in earlier reports.

**Figure 2:**
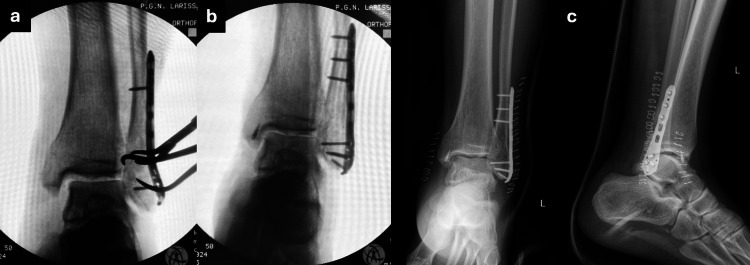
Intra-operative and post-operative X-rays. (a) Intraoperative radiological examination revealing medial joint widening despite fibula reduction (b) Complete restoration of ankle joint congruency after disengagement of the deltoid ligament which was reattached on the medial malleolus using a suture-anchor (c) Post-operative anteroposterior and lateral X-rays.

However, although the fibula was anatomically reduced and fixed by locking plate and screws and no instability of the distal tibiofibular joint could be noticed, image intensifier X-rays revealed significant widening of medial malleolus joint space and talus lateralization (Figure 2a). Deltoid ligament rupture and interposition were suspected, and the lesion was confirmed and repaired using a suture-anchor by medial approach (Figure 2b). New X-rays were satisfying, and surgical wounds were closed. A posterior ankle splint in a neutral position was applied.

**Figure 3:**
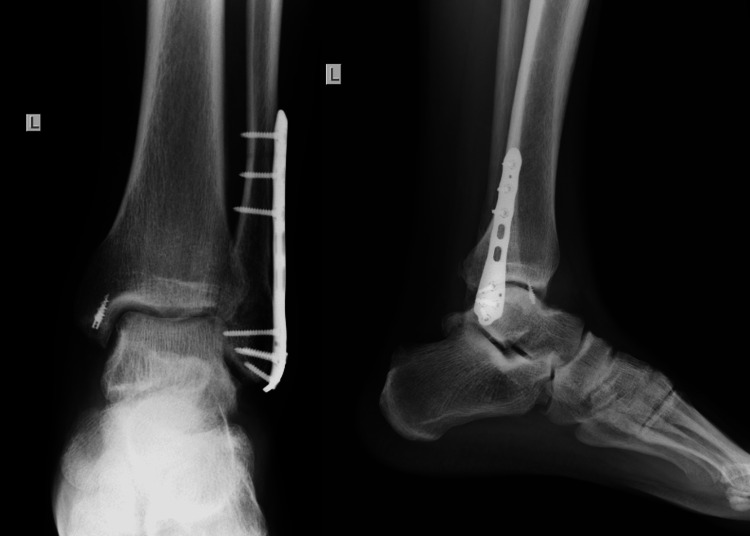
Three-month follow-up X-rays. Radiological examination in three-month follow-up.

Non-weight bearing was suggested for four weeks, mobilization of the ankle started after a month of removing the splint, and full-weight bearing was allowed after six weeks post-operatively. Rehabilitation of the patient was uneventful and radiological and functional outcomes were perfect in the three-month follow-up (Figure 3). 

## Discussion

Bosworth injuries are rare and challenging regarding both diagnosis and management. According to the literature, there are just a little more than one hundred cases recorded and such rarity, accounting for less than 2% of ankle fractures, carries the risk of delayed diagnosis or even misdiagnosis [2,5,7]. A rotational mechanism of ankle injury causes dissociation of the tibia and fibula resembling Galeazzi fracture-dislocation of the forearm. Malleoli (medial, lateral, or even posterior) may be fractured and according to the Danis-Weber classification, the vast majority of cases with fibula fracture are of type B while type A injuries are less than 1% [5].

X-ray imaging examination can be diagnostic as long as there is high awareness and good knowledge of this particular type of injury [1,2]. Though lateral view often reveals the posterior displacement or angulation of the fibula and the dislocation of the talus, an increased overlap of distal tibia and fibula may be marked in the anteroposterior X-rays. The “axilla sign,” characterized by cortical radiodensity of the medial tibial plafond due to internal tibial rotation, may be less frequently recognized in the anteroposterior view [4,5]. A CT scan can be used to reveal fibula displacement posterior to the incisura tibiae.

In our case, the misdiagnosed fibula dislocation led to persistent and worsening patient pain and discomfort despite ankle immobilization. This may serve as an additional warning sign when imaging fails to raise suspicion of a Bosworth injury. Repeated attempts of closed reduction and delayed restoration of the ankle anatomy increase the difficulty of intra-operative reduction of the fibula, and the risk of soft tissue complications, and compartment syndrome.

Regarding the management, fibula proximal segment fixed posterior dislocation with concomitant soft tissues (mainly tendons) interposition makes closed reduction manipulations highly ineffective [5]. Bosworth injuries are mainly treated by open reduction and internal fixation [8]. Such an anatomical abnormality makes the standard lateral malleolus approach risky, as commonly used landmarks may be challenging to identify. Moreover, tendons and nerves may be at the relative risk of entering abnormally into the surgical field, especially in obese patients. Furthermore, despite the meticulous posterolateral dissection, disengagement of the “locked” fibula may be tricky.

As soon as reduction has been achieved, the fibula can be fixed according to the morphology of the co-existing lateral malleolus fracture. The stability of the distal tibiofibular syndesmosis (DTFS) must be evaluated at this stage, as ligamentous injuries are common in Bosworth injuries. Though rupture or avulsion (from Tillaux-Chaput tubercle of the tibia or from Wagstaffe tubercle of the fibula) of the anterior tibiofibular ligament (ATFL) is common, complete rupture of the distal tibiofibular syndesmosis appears less often. However, the interosseous tibiofibular ligament may also be ruptured. Given the rotational mechanism of injury (involving either the fibula or tibia), stabilizing the distal tibiofibular syndesmosis with syndesmotic screws, after clearing any debris from the joint space, may be essential to ensure ankle joint congruence [8].

While fractures of the medial malleolus should be treated by internal fixation, insufficient reduction of the tibiotalar joint with the widening of the medial clear space or valgus tilt of the talus even after recognizing and reducing the fibula in a Bosworth injury, suggests a deltoid ligament rupture and interposition in the joint. The repair can be achieved using suture anchors [6,9]. In our case, a thick flap detached from the medial malleolus, deltoid ligament was removed from the joint and reattached using a suture anchor. Disengagement of the ligament allowed for the absolute restoration of the ankle anatomy which was confirmed by the image intensifier. 

Anatomical reduction of the distal fibula and the ankle joint is a prerequisite for a satisfying functional and radiological outcome. However, the injury itself and the operative treatment have potential risks of complications such as joint stiffness, skin necrosis, persistent pain, superficial peroneal nerve injury, avascular necrosis of the talus, and post-traumatic arthritis. The considerable complication rate, including the possibility of compartment syndrome, necessitates close peri-operative monitoring. On the other hand, the delay in diagnosis and surgical intervention as well as multiple failed attempts of closed reduction increase the risk of such complications. The acceptance of incomplete restoration of the anatomy of the ankle or failure to diagnose this rare complex injury leads to an unsatisfying outcome [6,7].

## Conclusions

Bosworth fracture-dislocation is a rare pattern of an ankle injury. The mechanism of such an injury induces multiple bone and ligamentous lesions apart from the locked dislocation of the fibula. Misdiagnosis or improper management results in escalating pain and discomfort that can be difficult for the patient to tolerate, ultimately leading to a poor functional outcome. Familiarity with the Bosworth injury is needed not only for the early diagnosis but for the operative treatment as well, which demands multiple interventions for the restoration of the anatomy and the functionality of the ankle.
